# Could the Oxidation of α1-Antitrypsin Prevent the Binding of Human Neutrophil Elastase in COVID-19 Patients?

**DOI:** 10.3390/ijms241713533

**Published:** 2023-08-31

**Authors:** Maura D’Amato, Monica Campagnoli, Paolo Iadarola, Paola Margherita Bignami, Marco Fumagalli, Laurent Roberto Chiarelli, Giovanni Stelitano, Federica Meloni, Pasquale Linciano, Simona Collina, Giampiero Pietrocola, Valentina Vertui, Anna Aliberti, Tommaso Fossali, Simona Viglio

**Affiliations:** 1 Department of Molecular Medicine, University of Pavia, 27100 Pavia, Italy; monica.campagnoli@unipv.it (M.C.); giampiero.pietrocola@unipv.it (G.P.); simona.viglio@unipv.it (S.V.); 2 Department of Biology and Biotechnologies “L. Spallanzani”, University of Pavia, 27100 Pavia, Italy; piadarol@unipv.it (P.I.); p.bignami98@gmail.com (P.M.B.); marco.fumagalli@unipv.it (M.F.); laurent.chiarelli@unipv.it (L.R.C.); giovanni.stelitano01@universitadipavia.it (G.S.); 3 Department of Internal Medicine and Medical Therapeutics, University of Pavia, 27100 Pavia, Italy; f.meloni@smatteo.pv.it (F.M.); valentina.vertui@gmail.com (V.V.); 4 Transplant Unit, IRCCS Policlinico San Matteo, 27100 Pavia, Italy; 5 Department of Drug Sciences, University of Pavia, 27100 Pavia, Italy; pasquale.linciano@unipv.it (P.L.); simona.collina@unipv.it (S.C.); 6 Division of Anesthesiology and Intensive Care 1, IRCCS Policlinico San Matteo, 27100 Pavia, Italy; an.aliberti@smatteo.pv.it; 7 Department of Anesthesiology and Intensive Care, ASST Fatebenefratelli Sacco, Luigi Sacco Hospital, University of Milan, 20157 Milan, Italy; tommaso.fossali@asst-fbf-sacco.it

**Keywords:** lung, α1-antitrypsin (AAT), human neutrophil elastase (HNE), COVID-19, SARS-CoV-2, broncho-alveolar lavage (BAL), liquid chromatography–mass spectrometry (LC–MS), neutrophils, oxidation, reactive oxygen species (ROS)

## Abstract

Human neutrophil elastase (HNE) is involved in SARS-CoV-2 virulence and plays a pivotal role in lung infection of patients infected by COVID-19. In healthy individuals, HNE activity is balanced by α1-antitrypsin (AAT). This is a 52 kDa glycoprotein, mainly produced and secreted by hepatocytes, that specifically inhibits HNE by blocking its activity through the formation of a stable complex (HNE–AAT) in which the two proteins are covalently bound. The lack of this complex, together with the detection of HNE activity in BALf/plasma samples of COVID-19 patients, leads us to hypothesize that potential functional deficiencies should necessarily be attributed to possible structural modifications of AAT. These could greatly diminish its ability to inhibit neutrophil elastase, thus reducing lung protection. The aim of this work was to explore the oxidation state of AAT in BALf/plasma samples from these patients so as to understand whether the deficient inhibitory activity of AAT was somehow related to possible conformational changes caused by the presence of abnormally oxidized residues.

## 1. Introduction

The finding of high levels of human neutrophil elastase (HNE) in both nasopharyngeal swabs and in the peripheral blood of patients infected by COVID-19, as well as the fact that this protease may be involved in SARS-CoV-2 virulence (the virus, in fact, requires the cleavage of the spike protein to enter host cells), means that HNE plays a pivotal role in lung infection [1,2,3,4]. Previous studies have shown the significant contribution of this serine protease secreted by neutrophils to lung tissue destruction in several pulmonary diseases, which is heavily responsible for elastin degradation [5,6,7]. Overexpression of HNE and pro-inflammatory cytokines generates a hypoxic environment at the level of lung parenchyma, the consequence of which is the release of high concentrations of reactive oxygen and nitrogen species (ROS and RSN, respectively) that are very harmful to lung tissue. Given this role, it can be assumed that if not suitably neutralized by a specific anti-protease, HNE activity would be heavily deleterious to the lung. In healthy individuals, HNE activity is balanced (in a 1:1 stoichiometric ratio) by α1-antitrypsin (AAT), a 52 kDa glycoprotein which is mainly produced (70–80%) and secreted by hepatocytes [5]. AAT specifically inhibits HNE by blocking its activity through the formation of a stable complex (HNE–AAT) in which the two proteins are covalently bound. AAT is responsible for more than 90% of the anti-proteinase activity in human serum, the remaining 10% being provided by β-2 macroglobulin. The proof that the balance between proteases and antiproteases is one of the most important aspects of lung homeostasis is provided by the onset of a disorder (α1-antitrypsin deficiency, AATD) wherein AAT circulating in the organism is missing/decreased [4]. AAT is not only a highly specific inhibitor of HNE but, as has recently been suggested, it is able to neutralize a broad range of other serine proteases and also different classes of proteases, including, for example, cathepsin G, proteinase-3, metalloproteases, myeloperoxidase, and cysteine-aspartic proteases [8,9,10,11,12,13,14]. Moreover, a potential protective role of AAT in COVID-19 defense has been hypothesized by some authors based on the ability of this inhibitor to prevent the entry of SARS-CoV2 mediated by host transmembrane protease serine-type 2 [15,16]. These peculiar features mean that AAT plays a key role in regulating inflammatory response. The formation of the AAT–HNE complex in real samples was demonstrated, for the first time, in the bronchoalveolar lavage fluid (BALf) of patients with bronchiolitis obliterans syndrome (BOS) [4]. Unambiguous identification of this complex, whose amount correlated with the level of lung inflammation, was achieved by means of mono-dimensional sodium dodecyl sulphate polyacrylamide gel electrophoresis (1D, SDS-PAGE) coupled to liquid chromatography–mass spectrometry (LC–MS) [4]. Surprisingly, the same approach applied to the analysis of BALf samples from COVID-19 patients did not allow us to observe traces of the complex, and no information was provided as to which of the two components (HNE and AAT) was responsible for the failure to form it [17]. However, the detection of HNE activity in these samples led the authors to hypothesize that potential functional deficiencies, if any, should necessarily be charged to AAT. According to this assumption, possible structural modifications of AAT could greatly diminish its ability to inhibit neutrophil elastase, thus reducing lung protection. In particular, modifications of methionine(s) (specifically at positions 351 and 358) caused by oxidants released by inflammatory cells are of great importance in this process since these residues play a pivotal role in establishing the proteinase–inhibitor complex. In fact, the cited post-translational modification leads to the formation of oxidized forms of AAT that not only have minimal or no anti-elastase activity but have also been shown to be converted into proinflammatory stimuli [18,19,20,21].

Based on this evidence, we intended to explore the oxidation state of AAT in BALf/plasma samples from COVID-19 patients with the aim of understanding whether AAT is actually oxidized and, in this case, whether oxidized AAT can no longer bind to HNE. In addition, we tested whether other proteins would replace HNE in their binding to oxidized AAT.

## 2. Results

### 2.1. Protease/Antiprotease Balance in BALf Samples Added of Exogenous AAT

**Figure 1:**
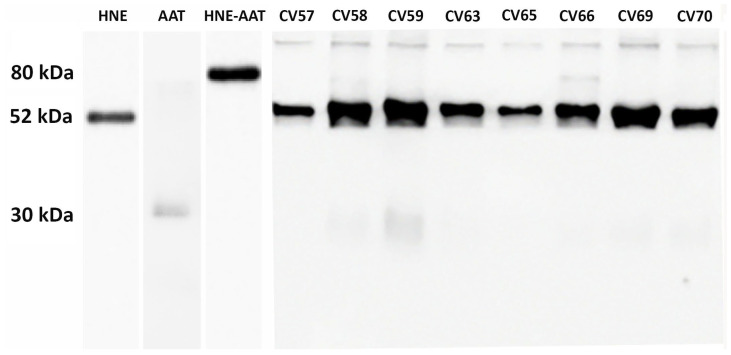
Western blot of BALf from COVID-19 samples supplemented with exogenous AAT upon treatment with anti-AAT and anti-HNE antibodies (lanes 4 to 10). In lanes 1, 2, and 3 are reported AAT alone, HNE alone, and the complex formed between HNE and AAT, respectively.

As previously demonstrated [17], endogenous AAT in COVID-19 patients is unable to inhibit the HNE activity through the formation of the HNE–AAT complex. Thus, the effect of mimicking the therapy in use for AAT-deficient patients [22] by adding exogenous AAT was explored. Each BALf sample was spiked with 0.4 μg of standard AAT, incubated for 15 min, and submitted to SDS-PAGE followed by Western blot analysis with specific anti-AAT and anti-HNE antibodies. The results obtained from eight samples (CV57 to CV70, representative of all others) are shown in Figure 1. The SDS-PAGE profiles of other BALf samples investigated were very similar to those shown in Figure 1. An example is reported in Figure S1 of Supplementary Materials.

It can be noticed that just as the bands corresponding to AAT and HNE—52 kDa and 30 kDa, respectively (despite being the latter present in the form of light bands)—were found in all samples, likewise, the band of AAT–HNE complex (usually identified at about 80 kDa) is absent in all samples (with the exclusion of samples CV 58 and CV 66, in which light bands could be observed). On the other hand, all samples show a number of thin bands in the molecular weight range of 180–250 kDa.

**Figure 2:**
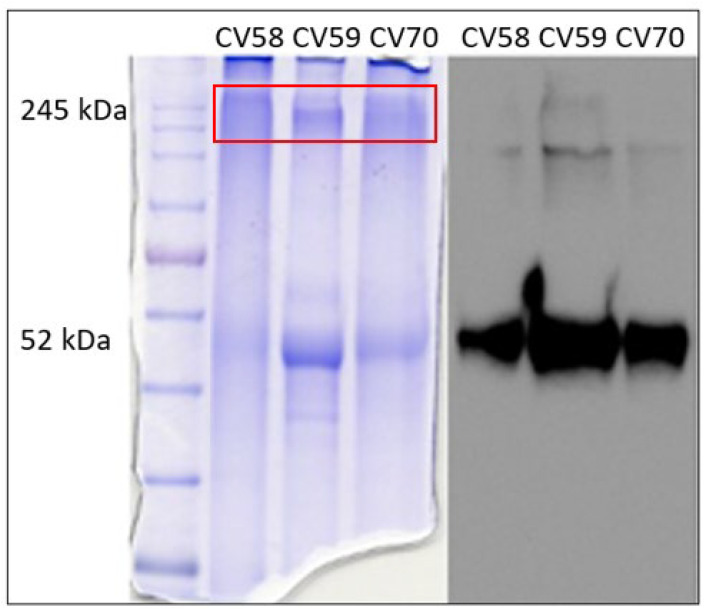
SDS-PAGE on 8% running gel. Gel stained with Coomassie blue-containing picric acid (on the **left**) and gel submitted to Western blotting with anti-AAT antibodies (on the **right**). The box in red indicates the region of the gel from which proteins were extracted and analyzed via LC–MS.

To gain insight into these bands, three samples (CV58, CV59, and CV70) showing high molecular weight bands with marked intensity were chosen as representative specimens. To improve the resolution of the bands, each sample was further run on SDS-PAGE using a running gel with lower density (8%). At the end of the run, the gel was vertically divided into two parts, one of which submitted to Western blotting with anti-AAT antibodies (Figure 2, right), and the other was stained with Coomassie blue-containing picric acid (Figure 2, left).

The bands corresponding to high molecular weight (identified by the red box in Figure 2, left) were excised, extracted from the gel, digested with trypsin, and analyzed via LC–MS/MS. The results are summarized in Table 1.

The identification of immunoglobulins under these bands (and the absence of the band corresponding to the HNE–AAT complex) led us to speculate that in COVID-19 samples, there was a sort of “preference” in AAT for complex proteins other than the target one (HNE). To better investigate this unusual behavior, AAT was purified from the plasma and BALf of COVID-19 patients and submitted to structural analyses.

### 2.2. Purification of AAT from Plasma and BALf Samples

**Figure 3:**
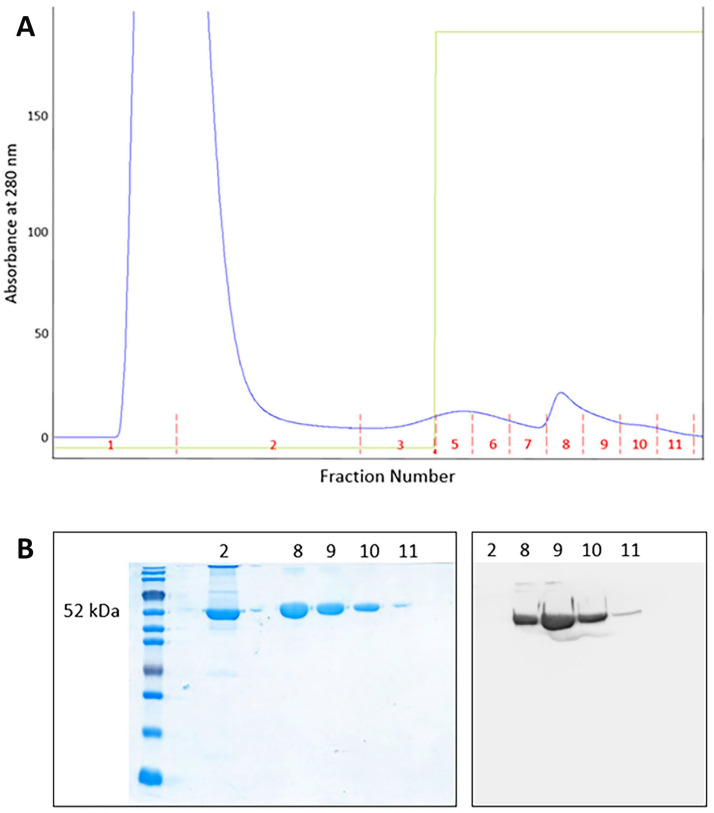
(**A**) Chromatographic profile showing the purification of AAT from a plasma sample. Fractions 8 to 11 were collected and submitted to SDS-PAGE; (**B**) SDS-PAGE of fractions 2 and 8 to 11 (**left**) and Western blotting (**right**) of fractions 8 to 11 obtained after purification of AAT from a plasma sample.

AAT was purified from both plasma and BALf samples of SARS-CoV-2 patients through affinity chromatography. The profile obtained from a plasma sample (representative of all plasma specimens analyzed) is depicted in Figure 3A. The fractions 8 to 11 were collected, desalted, and subjected to SDS-PAGE followed by Western blot analysis with anti-AAT antibody (Figure 3B). The purification profile of AAT from BALf samples resulted very similar to that reported in Figure 3A.

Fractions containing purified AAT (fractions 8 to 10 in Figure 3A) were pooled, concentrated, and stored for further analysis.

### 2.3. Analysis of AAT by HPLC

**Figure 4:**
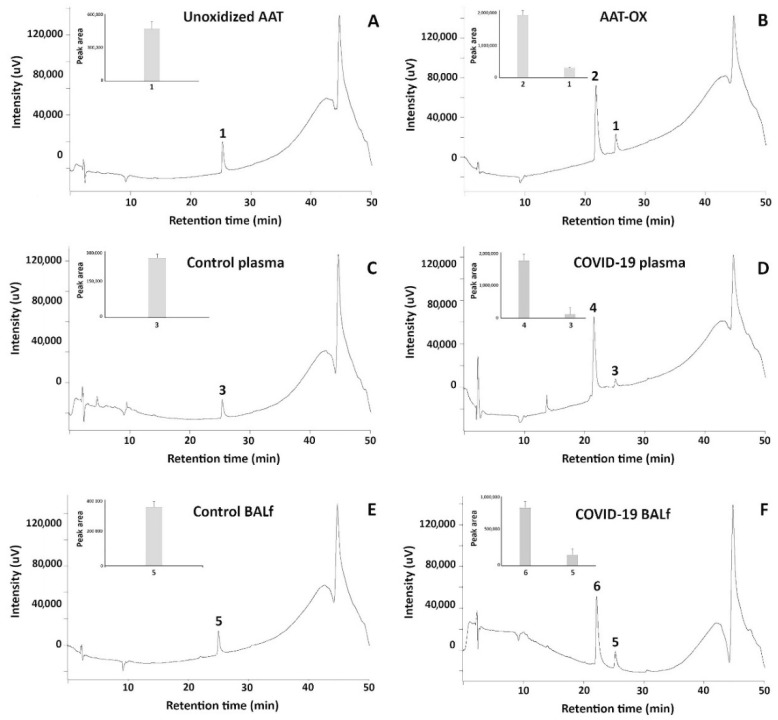
RP-HPLC profiles of standard unoxidized AAT and standard in vitro oxidized AAT ((**A**,**B**), respectively); of native AAT purified from the plasma of control and COVID-19 patients ((**C**,**D**), respectively); and of native AAT purified from the BALf of control and COVID-19 patients ((**E**,**F**), respectively).

To acquire insights into the status of AAT in real samples, aliquots of this inhibitor purified from plasma and BALf (of healthy and COVID-19 patients) as mentioned above, were loaded on RP-HPLC in parallel with normal and in vitro oxidized standard AAT. As far as the standard AAT is concerned, Figure 4 shows that while non-oxidized protein is characterized by a single peak eluting at 24.30 ± 0.5 min (peak 1, Figure 4A), the in vitro oxidized sample contains an additional peak eluting at 21.20 ± 0.4 min (peak 2, Figure 4B). AAT from the plasma of healthy individuals shares the same profile of standard AAT since a single peak with elution time 24.50 ± 0.3 min (peak 3, Figure 4C) is clearly detected. Interestingly, the pattern of AAT from the plasma of COVID-19 patients perfectly overlapped with that of oxidized standard AAT, as the presence of a second peak with an elution time of 21.15 ± 0.4 min can be appreciated (peak 4, Figure 4D). As expected, the profiles of AAT purified from BALf of control and COVID-19 patients (Figure 4E,F, respectively) were virtually identical to those from plasma.

Based on the peak areas, it could be observed that the appearance of peaks 2, 4, and 6 came at the expense of peaks 1, 3, and 5, respectively (see insets of the panels).

As different elution times meant different interaction with the hydrophobic stationary phase, to understand the difference between the proteins under the peaks, all of them were collected, and the material was analyzed via LC–MS. The results are shown in Table 2.

### 2.4. Sequence Alignment

**Figure 5:**
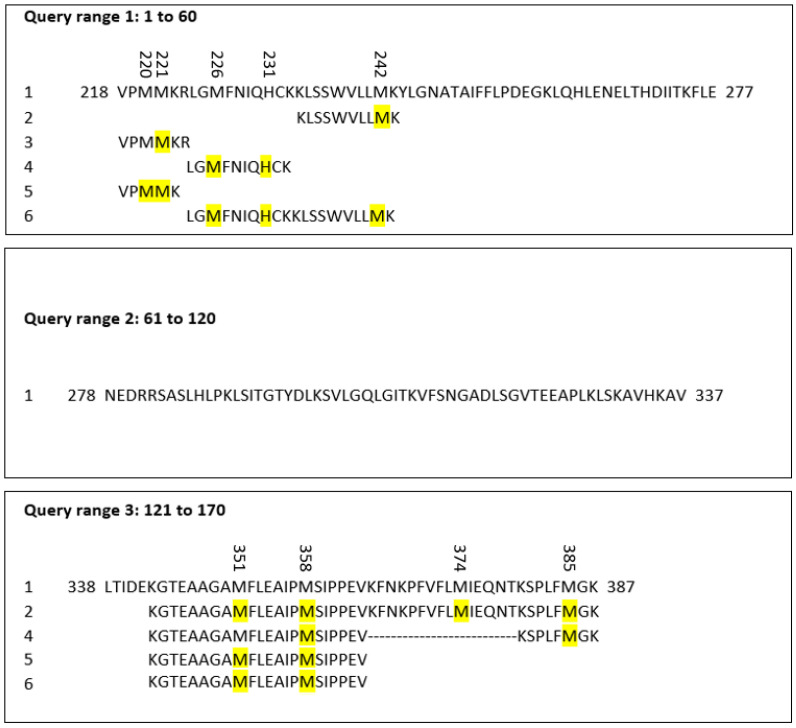
Alignment of AAT sequences showing the oxidized residues (marked in yellow) identified in proteins purified via HPLC and analyzed via MS.

The peptide sequences of AAT containing the oxidized residues found by MS were aligned using pBlast software 2.14.1 (https://blast.ncbi.nlm.nih.gov/Blast.cgi?PAGE=Proteins; accessed on 15 March 2023) and compared by performing the match with the AAT query, whose FASTA format was taken from Uniprot (https://www.uniprot.org/; accessed on 15 March 2023). The query used was the following: sp|P01009|A1AT_HUMAN Alpha-1-antitrypsin OS = Homo sapiens OX = 9606 GN = SERPINA1 PE = 1 SV = 3 Query ID: lcl|Query_45999 Length: 418. The oxidized residues are highlighted in yellow in Figure 5.

### 2.5. Molecular Dynamics of Chemically Oxidized AAT

**Figure 6:**
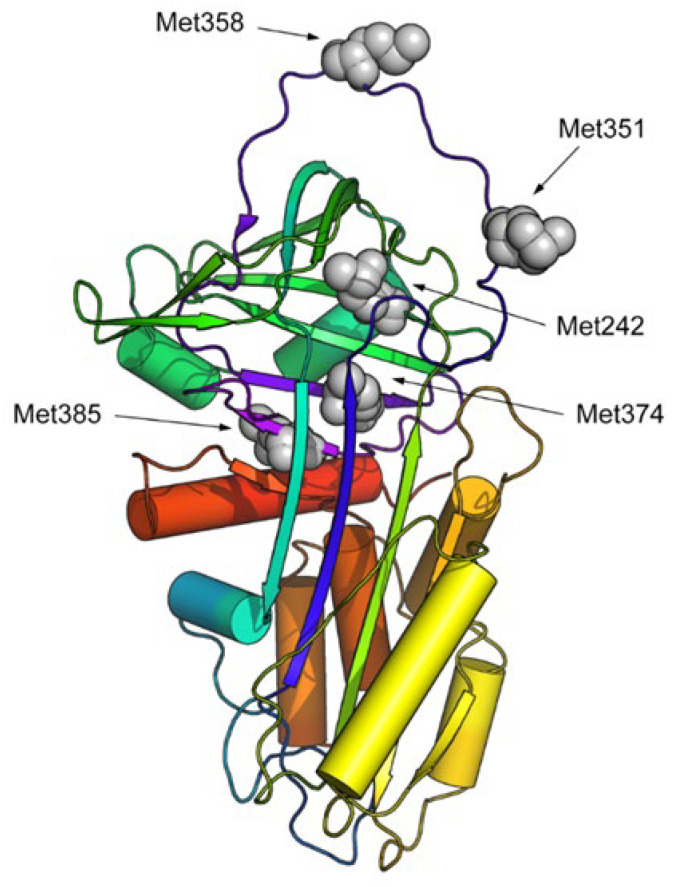
Cartoon representation (in rainbow colors) of the 3D structure of human AAT. Oxidized methionine residues are highlighted via grey CPK representation.

Molecular dynamics (MD) was used to assess whether the oxidation of methionine key residues in AAT, as observed in COVID-19 patients, can lead to a remarkable conformational change in the protein structure, potentially explaining the loss of the protein activity. To analyze the effect of oxidation on the stability and conformational alteration of the AAT structure, all the methionine residues identified as having been oxidized to methioninesulfoxide in the chemically induced oxidized AAT were considered, i.e., Met242, Met351, Met358, Met374, and Met385 (Figure 6).

**Figure 7:**
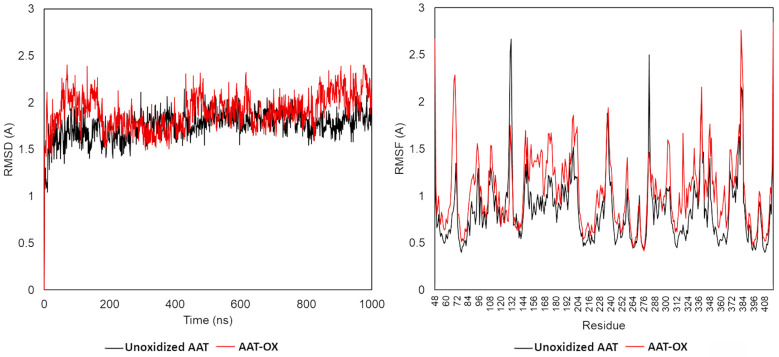
Molecular dynamics (MD) trajectory analysis of unoxidized and oxidized AAT. Root mean square deviation (RMSD, **left**) and root mean square fluctuation (RMSF, **right**) of Cα atom coordinates throughout 1 μs of MD for the unoxidized (black line) and AAT-ox (red line).

The root mean square deviation (RMSD), root mean square fluctuation (RMSF), and secondary structure elements (SSE) were analyzed in both native and oxidized AAT (AAT-OX) model at 1000 ns. The C-alpha RMSDs of both proteins during the 1000 ns dynamic are reported in Figure 6. The small deviation of the protein backbone atoms during 1 microsecond of simulation indicates that all methionine oxidations were tolerated, with an overall root mean square deviation <2.2 Å in both protein structures. In detail, the RMSD for the wildtype protein started at 1.4 Å at 0 ns and reached equilibrium around 50 ns, maintaining the RMSD fluctuation in the 1.25–2.0 Å range. This indicates that the unoxidized protein maintained a relatively stable conformation during the simulation. In contrast, the RMSD analysis of AAT-OX demonstrated a slightly greater fluctuation in comparison to the unoxidized model (Figure 7, left). The RMSD of the AAT-OX model displayed a steady increase up to 2.0–2.4 Å in the first 150 ns of simulation. Subsequently, within the following 250 ns, it experienced a sudden decrease from 0.5 Å to 0.75 Å. However, it started to rise again at 450 ns up to 2.0 Å, before exhibiting fluctuations beyond 2.0 Å during the last 200 ns of the simulation. The higher oscillations observed in the AAT-OX compared to the unoxidized AAT suggest a slight increase in flexibility and conformational fluctuations in the AAT-OX. These fluctuations indicate a structural rearrangement, possibly attributed to the presence of methionine oxidations. However, it is important to note that the overall small deviations observed in the protein backbone atoms (RMSD ± 1 Å throughout the entire simulation) are not significant enough to cause destabilization of the protein structure. This suggests that the structural integrity of both proteins was maintained, and the presence of methionine oxidations in the AAT-OX was tolerated.

To investigate in greater depth whether the observed slight increase in the flexibility of the AAT-OX over the unoxidized protein might be the consequence of protein conformational changes and to identify the regions mainly affected, we conducted an RMSF and secondary structure elements (SSE) analysis. The RMSF value over the entire simulation showed a higher fluctuation in the oxidized AAT over the native protein (Figure 7, right). Besides the N- and C- termini, for which a high fluctuation (>2.5 Å) was expected, the most fluctuating regions corresponded to the most exposed and less structured portion of the protein, such as the reactive center loop (RCL, residues 342–364), the loop connecting the 5A and 6A beta-sheets (residues 306–331), and the loop connecting helix-E with the 1A beta-sheet (residues 121–132). Additionally, a notable RMSF value exceeding 1 Å was observed for the short loop connecting helix-A with helix-B (residues 43–49), which may be attributed to the oxidation of Met385 in the C-peptide. The oxidation of Met385 to methionine sulfoxide introduces a more polar moiety, leading to a destabilization in the hydrophobic environment of helix-A. This destabilization causes increased fluctuations in this specific region of the protein, contributing to the observed higher RMSF value. Conversely, no significative variation in the fluctuations in the protein regions assembled to form and ordered secondary structure was observed in the AAT-OX over the unoxidized protein. The analysis of the secondary structure performed on both proteins confirmed this observation; indeed, no significative variation in the % of SSE was observed between unoxidized AAT and AAT-OX. Briefly, the native AAT counts for an average of 51.04% of the secondary structure elements (SSE) throughout the simulation is divided into 25.70% of helices and 25.34% of strands, whereas the AAT-OX counts for an average of 50.90% of SSE is divided into 24.93% of helices and 25.97% of strands.

**Figure 8:**
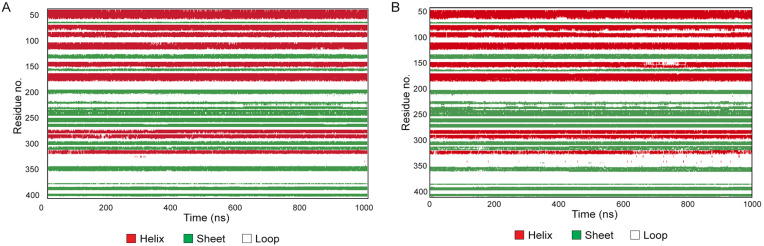
Secondary structure elements (SSE) analysis over the 1000 ns molecular dynamics for unoxidized AAT (**A**) and AAT-OX (**B**).

The plot in Figure 8 monitors each residue and their SSE assignment over the entire simulation, revealing that only the residues of the SSE in connection with the loops are affected by the fluctuation of the less structured portion, as already observed in the RMSF analysis, albeit without destabilizing the secondary structure. On the contrary, the SSE that participated in the rearrangement of the protein conformation that occurs during the anti-protease activity of the protein remained well-conserved.

### 2.6. In Silico Prediction of AAT Forms Purified from Real Samples

Using the Maestro software (Schrödinger Maestro 9.0), the AAT structures containing the oxidized residues detected through MS were visualized in silico. The structures of standard AAT unoxidized AAT/AAT-OX and AAT purified from the plasma and BALf of control/COVID-19 patients are compared in Figure S2 (Supplementary Materials).

As shown, only three of the oxidized residues are present on the alpha-helix and the RCL, while the others are in less exposed portions of the protein. The energy analysis of the structures (see Table S1 of Supplementary Materials) showed that the potential energy is mostly unchanged among the different oxidation structures, while the internal energy structures slightly differed, depending on the number and position of the oxidized amino acids.

### 2.7. Circular Dichroism

Based on the results shown above, the CD spectra of standard AAT (normal and in vitro oxidized) were compared to those of the purified proteins from real COVID-19 samples (from both plasma and BALf) in order to investigate the effect of the oxidation on the secondary structure of the protein.

**Figure 9:**
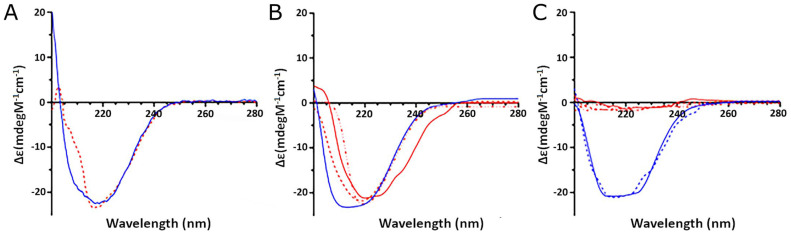
Circular dichroism analysis of AAT extracted from different samples: (**A**) CD spectra of recombinant AAT untreated (blue line) and after oxidation (red line); (**B**) CD spectra of AAT extracted from plasma samples of control (blue line) and COVID-19 (red lines) subjects; (**C**) CD spectra of AAT extracted from BALf samples of control (blue lines) and COVID-19 (red lines) subjects.

As shown in Figure 9A, the in vitro oxidized AAT (red line) exhibited a consistent change in the CD spectrum compared to the unoxidized protein (blue line). In detail, a significant decrease in the positive peak at 200 nm and a loss of the peak at 208 nm were observed. The spectra deconvolution allowed us to estimate that treatment with H_2_O_2_ led to a significant loss in alpha-helical content, thus confirming that oxidation affects the general folding of the protein.

The CD analysis of the AAT purified from the plasma from one control (blue line) and three representative COVID-19 (red lines) individuals (Figure 9B) also demonstrated that the oxidation occurring in the COVID-19 samples led to a significant loss of alpha-helical content, thus confirming its effect on the folding of the protein.

Even more remarkable were the effects of oxidation on the AAT extracted from the BALfs of COVID patients (Figure 9C, red lines), which appear to have completely lost folding, while the protein from control BALfs (blue lines) displayed an alpha-helical content similar to that of the standard protein.

## 3. Discussion

Under the assumption that AAT is responsible for the protection of lung tissue against the proteolytic activity of HNE released from neutrophils through an irreversible reaction leading to the formation of the AAT–HNE complex, great was our surprise upon noticing the lack of this complex in both the BALf and plasma from COVID-19 patients. The first reading of this result was that the amount of circulating AAT was not enough to let us detect the complex formation. To explore this hypothesis, scalar amounts of exogenous AAT were added to the samples, somehow mimicking the current “augmentation” therapy in use for the treatment of patients affected by the genetic disease known as AAT deficiency [23,24]. The failure of this test and the observation that AAT reacted preferentially with heavy chains and C regions of different immunoglobulins (predominantly IgG) led us to hypothesize that due to the peculiar physiological conditions, a possible structural modification of AAT made it appear as an antigen to the antibodies present in the blood of these patients. Thus, it has been speculated that the strongly oxidative environment related to SARS-CoV-2 infection could somehow be responsible for the AAT activity impairment. It is worth remembering that the massive presence of neutrophils brings the release of ROS and the consequent growth of an extreme environment that might have oxidized AAT, thus depriving this inhibitor of its ability to complex HNE. Light was shed on this scenario by the concurrent finding of native and oxidized forms of AAT in samples from COVID-19 patients observed to be overlapping the chromatographic profiles of purified AAT from real samples with those of standard AAT oxidized in vitro. This first hint of this hypothesis was confirmed by the alignment of peptides containing oxidized residues in the various samples, although interpretation of these data was somewhat questionable. First, the finding of “naturally occurring” oxidized residues (Met220 and Met221 in plasma and Met221 in BALf) in the AAT of “controls” was not such a surprise since samples necessarily came from subjects from whom these fluids (specifically BALf) could be taken. Thus, our “controls” were smokers or individuals affected by pulmonary disorders (i.e., BOS) who, while being non-COVID-19 patients, were not strictly “healthy”. The fact that the mentioned residues were not involved in the active site accounted for the ability of AAT to completely inhibit HNE, as previously proven [4]. As far as the overall situation of the COVID-19 samples was concerned, it appeared different from that of the controls not only in terms of number of Met residues oxidized but also in terms of their sequence distribution. The most interesting finding was the identification among the three oxidized Met residues in plasma (Met226, Met358, and Met385) of a Met residue (Met358) belonging to the active site of the protein. This residue was not identified among the two Mets (Met226 and Met242) found in BALf. The fact that the Met residues mentioned above were in the same as those identified in standard AAT oxidized in vitro was indirect evidence for the assumption that oxidation resulting from SARS-COV-2 infection could have occurred on AAT. Great emphasis was placed on the finding, in both plasma and BALf, of an oxidized His residue (His 231). In fact, if oxidation at the level of Met residues is a very common event when a protein lies in an oxidizing environment, the oxidation of histidines is less common. Apparently, even more controversial was the absence of this event in AAT oxidized in vitro. However, as already known, neutrophil granulocytes express several enzymes, including myeloperoxidases, involved in the control of host infections [2]. Once released from granules, these molecules impact the inflammatory response and can cause severe damage to the host as they infiltrate around the capillaries in the lungs. This is what has been observed in SARS-CoV-2 patients [25]. It is well known that endoperoxides or hydroperoxides can be generated from singlet oxygen, which is yielded through various physical and biochemical processes, including myeloperoxidase-mediated reactions [24]. In particular, the addition of O_2_ to His forms short-lived endoperoxides that can open to generate hydroperoxides at the ring position. Since the reaction of singlet oxygen with these side chains is rapid and selective, the formation of hydroperoxides can be very efficient. The existence of this mechanism would explain why oxidized His was only found in real samples and not in samples oxidized in vitro with hydrogen peroxide. Based on these findings, we have wondered how the oxidation of these residues, in particular methionines, could impact the stability and conformational alterations in the AAT and, consequently, be responsible for the failure to form the complex with HNE.

As a first step, molecular dynamics (MD) simulations have been carried out on unoxidized standard AAT and in vitro oxidized standard AAT (AAT-OX). RMSD and RMSF analyses were performed to assess the protein dynamics and identify regions affected by oxidation. Additionally, the secondary structure elements (SSE) were analyzed to evaluate any structural changes. The RMSD analysis revealed that both the unoxidized AAT and the AAT-OX maintained overall structural stability throughout the 1000 ns simulation. The small deviations observed in the protein backbone atoms indicated that the methionine oxidations were well-tolerated, as the RMSD values remained in the ±1 Å for both protein structures. The unoxidized AAT exhibited a relatively stable conformation throughout the simulation, whereas the AAT-OX demonstrated slightly greater fluctuations compared to the unoxidized protein, indicating a potential increase in flexibility and conformational changes induced by the oxidation. To gain further insights into the structural alterations, RMSF analysis was performed. RMSF provides information about the local fluctuations of individual residues throughout the simulation. The RMSF values showed that the oxidized AAT exhibited slightly higher fluctuations compared to the native protein. The regions experiencing the most significant fluctuations were primarily located in the exposed and less structured portions of the protein, with the main effect on the reactive centre loop (RCL) and in the other less structured portion of the protein. Conversely, the regions involved in the formation of ordered secondary structures, such as helices and strands, did not exhibit significant fluctuations in the oxidized AAT compared to the native protein. The analysis of secondary structure elements confirmed this observation, as the percentage of SSE remained largely unchanged between unoxidized AAT and AAT-OX. Further examination of the SSE distribution throughout the simulation using a residue-wise plot revealed that the fluctuations primarily affected the residues in connection with the aforementioned loops and less structured regions. However, these fluctuations did not lead to destabilization or significant alterations in the secondary structure elements involved in the protein’s anti-protease activity.

Overall, these findings suggest that the loss of functional activity observed in AAT-OX, particularly in COVID-19 patients, might not be due to the overall alterations in the secondary structure resulting from methionine oxidation. Instead, the functional impairment is likely attributed to specific oxidized residues, namely, Met358 and, to a lesser extent, Met351.

Met358 and Met351 are located within the RCL of AAT. In particular, Met358 is the P1 residue that directly participates in the catalytic cleavage of the peptide bond, facilitated by trypsin or other peptidases, such as HNE. The oxidation of Met358 may disrupt the recognition of the Met358-Ser359 peptide bond by the catalytic binding site of HNE, leading to the complete loss of AAT inhibitory activity. The importance of Met358 and its oxidation in the functional activity of AAT is highlighted by the fact that the oxidation of this specific residue plays a significant role in the observed loss of the inhibitory activity [19,26,27]. Although the oxidation of other methionine residues may contribute to the overall structural changes and flexibility observed in the oxidized AAT, they do not directly affect the inhibitory function of the protein. The presence of other oxidized methionine residues may induce conformational alterations and contribute to the overall stability and flexibility of the protein, but they are not directly responsible for the loss of activity, at least in the chemically induced AAT-OX.

As a second step, an in-silico prediction of structural models was applied to AAT purified in real samples to identify the molecule portions potentially susceptible to oxidation. Interestingly, also in this case, among the oxidized residues, only three of them were present on the alpha-helix and the RCL, while the others were found in the less exposed portion of the protein. Furthermore, from the energy analysis of the structures (Table S1 of Supplementary Materials), it appeared that while the potential energy remained unchanged among the different oxidation structures, the internal energy of the structures (as assessed by Prime) differed slightly depending on the number and position of the oxidized amino acids.

These differences in energy between structures are explained by the expected change in position of the oxidized amino acids to accommodate the new, albeit slight, local electronegativity added by oxygen to the side chain (see Figure S2 in Supplementary Materials). This analysis may be indicative of abnormal protein behavior. Indeed, a positive energy change can affect the stability of the structure, albeit locally, via a “domino effect” on the remainder of the tertiary structure over time, as a less negative free energy (ΔG) value is indicative of a non-spontaneous process. Conversely, a negative energy change is optimal from the point of view of the spontaneous folding reaction but may induce local stiffening that could possibly constrain any conformational changes in the protein due to the electrostatic bonds between oxygen and hydrogen in the closed side chains. However, the two largest differences in energy are observed in the COVID-19 BALf and plasma samples, probably because most, if not all, of the oxidation is in the proximity of the reactive loop compared to the other samples, in which the oxidations are located elsewhere in the structure.

Therefore, from the data obtained via molecular dynamics and in silico models, it can be hypothesized that the loss of functional activity in AAT-OX, as observed in COVID-19 patients, is primarily attributed to the oxidation of Met358, with Met351 also playing a minor role. These findings emphasize the significance of specific residue oxidation within the RCL region and its impact on the interaction with HNE.

To verify this hypothesis, unoxidized and AAT-OX and purified AAT from real samples were submitted to CD analysis. Surprisingly, notable differences were observed in the secondary structure of AAT extracted from both plasma and BALf samples of COVID-19 patients, compared to the AAT from controls. In fact, the CD spectra of AAT from plasma samples were very similar to that of the AAT-OX, with a consistent decrease in alpha-helical content, thus confirming the effects of oxidation on folding. The differences emerged as more dramatic in AAT from the BALf of COVID-19 samples, which, in contrast to what was deduced from the analysis of in silico models, appeared completely unfolded. However, it should be noted that due to the harsher conditions in the inflamed lung, other factors, in addition to the strongly oxidative environment, may have contributed to the loss of protein folding.

Although the results appear intriguing, it is imperative to emphasize that these are merely preliminary analyses that will have to be validated with an in vitro experimental approach. However, it seems that oxidation may lead, at least to some extent, to the conformational changes seen in the AATs extracted from COVID-19 patient samples, which are likely responsible for the lack of complex formation between AAT and HNE. Further studies will be carried out to validate this hypothesis and to understand the discordance between the data obtained via in silico models and CD analysis.

## 4. Materials and Methods

### 4.1. Reagents

α1-Antitrypsin, lyophilized powder, from human plasma, acetonitrile (ACN), formic acid (FA), and all other analytical grade reagents were purchased from Sigma Aldrich (St. Louis, MO, USA).

All buffers used for chromatographic/electrophoretic experiments were prepared using double-distilled water obtained with a Millipore (Bedford, MA, USA) Milli-Q purification system. Bicinchoninic acid (BCA) protein assay kit was obtained from Thermo Scientific (Rockford, IL, USA).

Antibodies for detection of AAT, HNE, and secondary anti-mouse antibodies were obtained from Abcam (Cambridge, UK) and Thermo Scientific (Rockford, IL, USA).

### 4.2. Patients

Patients considered in this study were the same as those of a previous investigation. To obtain information concerning their demographic and clinical data, please see reference [17] of this report. In brief, patients with severe pneumonia (*n* = 33; mean age 59.66 ± 20) were admitted with positive SARS-CoV-2 polymerase chain reaction (PCR) tests to the intensive care units of two institutions: The Territorial Social-Health Agency (ASST Fatebenefratelli Sacco, Milan, Italy) or the Scientific Institute of Hospitalization and Treatment (IRCCS San Matteo Foundation, Pavia, Italy). The sample collection was approved by the Local Ethics Committee (Comitato Etico di Area 1, prot. 20100005334 as for Sacco and 20200046007 as for IRCCS San Matteo) and was undertaken in accordance with the ethical principles of the Helsinki declaration. Informed consent was collected from all participants or next of kin.

### 4.3. BALf and Plasma Collection and Processing

BALf was collected (from March 2020 to December 2021) from ICU patients at admission, usually before starting experimental treatment strategies. BALf was treated according to the guidelines during the first and second wave of COVID-19, with approved therapies as steroids and low-molecular-weight heparin, and during third wave, with tocilizumab. All procedures for the collection, transport, and preparation of the samples were carried out according to the restrictions and protocols required by Italian law.

BALf collection was performed as previously described [28]. Briefly, the distal tip of the bronchoscope was wedged into the middle lobe or lingular bronchus, and a total of 150 mL of warm sterile saline solution was subsequently instilled in five 30 mL aliquots, which were sequentially retrieved by gentle aspiration. The first aliquot collected (20 mL) was used for a series of analyses which included microscopic and cultural examination of common bacteria and fungi and direct/cultural investigations of respiratory viruses. The returned fluid from the second to the fifth aliquots was pooled and further processed as BALf. Cells were recovered by centrifugation at 1500 rpm for 10 min, and supernatants were divided into aliquots (30 mL each) which were stored at −80 °C immediately after processing, where they remained until use.

Plasma was collected from healthy controls and ICU patients at admission and stored at −20 °C until the moment of use.

### 4.4. BCA Protein Assay

The protein concentration of each sample was determined by means of the bicinchoninic acid (BCA) assay [29] and through the preparation of a calibration curve produced using a solution of standard bovine serum albumin (BSA; Sigma Aldrich, St. Louis, MO, USA) in a range of concentration between 5 and 25 µg/mL.

### 4.5. SDS-PAGE

An aliquot of each BALf sample (about 20 µg) was submitted to protein precipitation with trichloroacetic acid (TCA) according to Yvon et al. [30]. After centrifugation, the pellet was reconstituted in 10 µL of 50 mM Tris HCl, pH 8.3, containing 5% 2-mercaptoethanol, 2% SDS, 0.1% bromophenol blue (BPB), and 10% glycerol. Samples were incubated at 95 °C for 5 min and then loaded on gel slabs consisting of 5% stacking gel and 12.5% running gel. Electrophoresis was performed according to Laemmli [31], applying a voltage of 150 V for 1 h. Gels were stained with colloidal Coomassie G-250 according to Candiano et al. [32].

### 4.6. Western Blotting

Protein bands obtained as indicated in the above paragraph were transferred to a Millipore polyvinyl divinyl fluoride (PVDF) membrane (Billerica, MA, USA) using a trans blot (BioRad Laboratories, Segrate, Milan, Italy) turbo system. After 1 h incubation in 5% milk (diluted in phosphate buffer saline, PBS) and three washes with phosphate buffer saline containing 0.1% Tween 20 (PBST), the membrane was incubated overnight with AAT antibody (ab9400; Abcam, Cambridge, UK) at a 1:2500 dilution in 1% milk. The membrane was washed three times with PBST (10 mL), incubated with the secondary antibody, rabbit anti-mouse IgG H&L (HRP) (ab6728, Abcam), at a 1:5000 dilution in 1% milk in PBST for 1 h at room temperature. After washing the membrane again (three times), it was incubated with PBS in ECL Westar ηC Ultra (Cyanagen, Bologna, Italy) solution according to the provided protocol. The same procedure was applied for the identification of free and complexed HNE by using the anti HNE antibody (MA5-2548, Invitrogen, Waltham, MA, USA) at a 1:1000 dilution and secondary antibody anti-rabbit (ab6721, Abcam) at a 1:3000 dilution. All immunoblots were acquired with the Image Quant LAS 4000 analyzer (GE Healthcare, Chicago, IL, USA).

### 4.7. Purification of AAT from BALf and Plasma by Affinity Chromatography

Purification of AAT from BALf and plasma samples was performed via affinity chromatography using a Fast Protein Liquid Chromatography (FPLC) ÄKTA Prime (Pharmacia Biotech, Uppsala, Sweden) system equipped with a column (total volume 4 mL) packed with Alpha-1 Antitrypsin Select resin (Cytiva, 17547201, Uppsala, Sweden). Briefly, 2 mL of sample (BALf or plasma) were loaded into this column, and unbound material was washed out using a buffer consisting of 20 mM Tris-HCl, 150 mM NaCl, pH 7.4. Elution of bound proteins was obtained by means of a buffer containing 20 mM Tris-HCl, 2 M MgCl2, pH 7.4. Finally, the column was regenerated by fluxing 10 column volumes of PBS, pH 2.0. The flow rate was 2 mL/min throughout the entire process. Fractions containing purified AAT were pooled, concentrated in a Speed Vacuum concentrator (Thermo Fisher Scientific Inc., Southend-on-Sea, UK), and stored for further analysis.

### 4.8. In Vitro Oxidation of Standard AAT

Aliquots of standard AAT (approximately 10 mM) dissolved in 50 mM Tris-HCl buffer, pH 7.5, were added of scalar concentrations (1, 5, 10, and 50 mM, and 30% p/v) of freshly prepared hydrogen peroxide (H_2_O_2_) solutions. The final volume of each sample was 100 µL. Incubation was carried out for 18 and 24 h, respectively, in a thermoblock device fixed at 30 °C. The reaction was stopped by removing H_2_O_2_ from the solution via size exclusion chromatography using a PD 10 (Amersham Biosciences, Uppsala, Sweden) column. The recovered material (about 2 mL) was concentrated to 100 µL using a Speedvac system (Thermo Fisher Scientific Inc., Southend-on-Sea, UK).

### 4.9. Reverse-Phase High Performance Liquid Chromatography (RP-HPLC)

Native AAT, oxidized AAT, and AAT purified from plasma and BALf from control/COVID-19 patients were analyzed via RP-HPLC using a Jasco (Japan Spectroscopic, Tokyo, Japan) PU 980 HPLC system equipped with a C18 (Waters, Etten-Leur, The Netherlands) column (150 × 4.6 mm i.d.; 3.5 µm particle size). Separations were carried out by applying a linear gradient from 100% solvent A to 60% solvent B in 40 min. Solvent A consisted of water containing 0.05% trifluoroacetic acid (TFA), and solvent B of ACN containing 0.05% TFA. At the end of each run, the column was washed for 2 min with 100% solvent B and then re-equilibrated for 5 min with 100% solvent A. The flow rate was 0.8 mL/min. Unless otherwise stated, a 20 µL sample volume was injected into the column for each run. The analytes were monitored via UV absorption at 220 nm.

### 4.10. In Situ Digestion

Enzymatic digestion of proteins was performed as previously described [33]. Briefly, the selected bands were carefully excised from the gel, placed into Eppendorf tubes, broken into small pieces, and washed with 100 mM ammonium bicarbonate, pH 7.8, containing 50% acetonitrile (ACN) until complete de-staining was achieved. The gel pieces were then dehydrated by adding 200 µL of ACN until they became opaque-white in color. The acetonitrile was finally removed. Gel pieces were dried under vacuum for 10 min and then rehydrated by adding 75 µL of 100 mM ammonium bicarbonate, pH 7.8, containing 20 ng/µL sequencing grade trypsin (Promega, Madison, WI, USA). The digestion was performed overnight upon incubation of the mixture at 37 °C, and the resultant peptides were extracted from the gel matrix via three-step sequential treatment with 50 µL of 50% ACN, 5% trifluoroacetic acid (TFA) in water, and, finally, with 100% ACN. Each extraction involved 10 min of stirring followed by centrifugation and removal of the supernatant. All supernatants were pooled, dried, and stored at −80 °C until mass spectrometric analysis. At the moment of use, the peptide mixture was solubilized in 0.1% formic acid (FA).

### 4.11. In-Solution Digestion of Purified Proteins

A total of 100 µL of each AAT sample purified via HPLC were submitted to in-solution digestion with sequencing grade trypsin. A total of 90 μL of 100 mM NH_4_HCO_3_, pH 8.0, and 10 μL of 100 mM DTT in 100 mM NH_4_HCO_3_ were then added. Samples were incubated for 30 min at 60 °C to reduce disulphide bridges and then digested at 37 °C overnight by adding sequencing grade TPCK-trypsin (0.1 μg/μL), reconstituted in 50 mM acetic acid, in a final 50:1 ratio. Finally, 2 μL of FA were added to inactivate trypsin. Samples were then dried in Speedvac for 2 h at 30 °C, resuspended in 20 μL of 0.1% FA, and stored at −20 °C until mass spectrometric analysis.

### 4.12. Liquid Chromatography-Tandem Mass Spectrometry (LC–MS/MS) for the Identification of Oxidized Residues in AAT of BALf and Plasma Samples

All analyses were carried out on a liquid chromatography–mass spectrometry system (Thermo Finnigan, San Jose, CA, USA) consisting of a thermostated column, a surveyor autosampler controlled at 25 °C, a quaternary gradient surveyor MS pump equipped with a diode array (DA) detector, and a linear trap quadrupole (LTQ) mass spectrometer with electrospray ionization (ESI) ion source controlled by Xcalibur software 1.4 (Thermo Fisher Scientific, Waltham, MA, USA). Analytes were separated by RP-HPLC on a Jupiter (Phenomenex, Torrance, CA, USA) C18 column (150 × 2 mm, 4 µm, 90 Å particle size) using a linear gradient from 95% solvent A (0.1% aqueous FA) to 60% solvent B (ACN containing 0.1% FA) in 60 min. The flow rate was 0.2 mL/min. Mass spectra were generated in positive ion mode under constant instrumental conditions: source voltage, 5.0 kV; capillary voltage, 46 v; sheath gas flow, 40 (arbitrary units); auxiliary gas flow, 10 (arbitrary units); sweep gas flow, 1 (arbitrary units); capillary temperature, 200 °C; and tube lens voltage, −105 V. MS/MS spectra, obtained via collision-induced dissociation (CID) studies in the linear ion trap, were performed with an isolation width of 3 Th *m*/*z*. The activation amplitude was 35% of ejection RF amplitude, which corresponds to 1.58 V. Data processing was performed using Peaks studio 4.5 software.

### 4.13. Molecular Dynamics

The crystal structure of native alpha-1 antitrypsin was retrieved from the Protein Data Bank (PDB ID: 3NE4). Chain A was selected for molecular dynamic study. The 3D structure of the oxidized AAT (AAT-OX) was developed by manually modifying Met242, Met351, Met358, Met374, and Met385 into the corresponding methionine sulfoxide. Both native and AAT-OX were prepared for calculations using visual molecular dynamics (VMD). Missing bonds and atoms were fixed, and polar hydrogens and charges were added. In addition, water molecules were removed. GROMACS was used for MD calculation on an NVIDIA GeForce RTX 3070 Laptop GPU. The complex was solvated using a TIP3P water model and neutralized with Na+ counterions in a cubic box with at least 1 nm spacing from the ligand–protein complex. The complex was subjected to energy minimization using “steepest descent algorithm”. Further, the minimized systems were equilibrated in constant temperature and volume (NVT) and constant temperature and pressure (NPT) ensembles using a “relax model system” before the simulation. In the initial steps, the energy-minimized systems are simulated in the NVT ensemble with Brownian dynamics at 10 K temperature for 100 ps and 12 ps with restraints on solute heavy atoms. In NPT ensemble systems, no restraints were applied on heavy atoms at 10 K and 300 K temperatures for 12 ps and 24 ps, respectively. The fully equilibrated systems were finally subjected to 1000 ns unrestrained MD simulations with a time step of 2 fs in an NPT ensemble with 1 bar (pressure) and 300 K temperature. The production simulation with a total of 1000 frames was used for further MDS analysis. The root mean square fluctuation (RMSF) and root mean square deviation (RMSD) were calculated using toolkits of the GROMACS package.

### 4.14. Oxidized AAT Structures Building, Minimization and Energy Calculation

The oxidized AAT structures were drawn starting from Protein Data Bank (PDB) AAT crystal structure 1QLP with Bioluminate 4.9, a software that is part of the Schrödinger suit. A 1QLP structure was prepared according to the Protein Preparation Wizard process using the OPLS4 force field, maintaining the crystal symmetry and filling the missing loops using Prime, a bioinformatic tool that is part of the same suite. The obtained optimized structure was the starting point from which to obtain the different oxidized structures according to the MS data. The energy of every structure was minimized according to the OPLS4 force field, and relative energy was calculated with Prime with the standard solvation model VSGB. Finally, every structure was refined via hybrid Monte Carlo minimization for 20 steps at 310 °K to mimic human temperature. The process automatically recalculates the internal energy of the structure with Prime.

### 4.15. Analysis of the Secondary Structure of AAT by Far-UV Circular Dichroism

The secondary structure of the standard native and oxidized AAT, as well as that of selected BALf and plasma samples, was analyzed via circular dichroism (CD). Measurements were performed in the far-UV region using a Jasco J-1500 spectropolarimeter equipped with a 1 mm path cell. Scans were conducted between 200 and 300 nm at 100 nm/min speed, with a spectral bandwidth of 1 nm and a sensitivity of 20 millidegrees. CD spectra measurements were performed at 25 °C in 50 mM Tris-HCl, pH 7.5. The protein concentration was 0.5 µg/µL. The alpha-helical and beta-sheet content were calculated by applying the BestSel software (https://bestsel.elte.hu/index.php; accessed on 18 April 2023).

## 5. Conclusions

To the best of our knowledge, the information documented in this report constitutes the most comprehensive description of oxidation at the expense of AAT in the plasma/BALf of SARS-CoV-2 patients. Our results show that the addition of oxygen atoms to the AAT of these patients causes slight distortions to the structure, which are mainly appreciable in the outer loop responsible for the HNE attack and in the alpha-helix (highlighted in yellow). These distortions appear as slight modifications, and the entire structure is not heavily altered or unfolded. However, while oxidation alone appears to be only partly responsible for the conformational change of AAT, this minimal change should make an important contribution to the lack of complex formation between AAT and HNE.

## Figures and Tables

**Table 1:** List of proteins identified under the bands present in the region of the gel marked by the box in red in Figure 2.

**Accession**	**Description**	**Score** **(%)**	**Coverage (%)**
sp|P02768|ALBU_HUMAN	Serum albumin OS = Homo sapiens	99	14.61%
sp|P01857|IGHG1_HUMAN	Immunoglobulin heavy constant gamma 1 OS = Homo sapiens	99	20.61%
sp|P0DOX5|IGG1_HUMAN	Immunoglobulin gamma-1 heavy chain OS = Homo sapiens	99	16.04%
sp|P01859|IGHG2_HUMAN	Immunoglobulin heavy constant gamma 2 OS = Homo sapiens	97	3.99%
sp|P20758|IGHA1_GORGO	Immunoglobulin alpha-1 chain C region OS = Gorilla gorilla	90	2.27%

**Table 2:** LC–MS/MS analysis of peaks collected from RP-HPLC.

Peak	Accession Number	Mass	Score (%)	Coverage (%)	Description	Peptide	Mr	Start → End
1	sp|P01009|A1AT_HUMAN	46,737	99	14.83	Alpha-1-antitrypsin OS = Homo sapiens	IVDLVK	685.437	169 → 174
						SVLGQLGITK	1014.61	301 → 310
						LSITGTYDLK	1109.59	291 → 300
2	sp|P01009|A1AT_HUMAN	46,737	99	63.40	Alpha-1-antitrypsin OS = Homo sapiens	SPLF(OxM)GK	794.399	381 → 387
						KLSSWVLL(OxM)K	1219.7	234 → 243
						FNKPFVFL(OxM)IEQNTK	1870.965	366 → 380
						GTEAAGA(OxM)FLEAIP(OxM)SIPPEVK	2290.122	344 → 365
3	sp|P01009|A1AT_HUMAN	46,737	30	7.18	Alpha-1-antitrypsin OS = Homo sapiens	VPM(OxM)K	620.302	218 → 222
4	sp|P01009|A1AT_HUMAN	46,737	99	15.55	Alpha-1-antitrypsin OS = Homo sapiens	LG(OxM)FNIQ(OxH)CK	1221.56	224 → 233
						GTEAAGAMFLEAIP(OxM)SIPPEVK	2274.12	344 → 365
						SPLF(OxM)GK	794.399	381 → 387
5	sp|P01009|A1AT_HUMAN	46,737	99	64.11	Alpha-1-antitrypsin OS = Homo sapiens	VP(OxM)(OxM)KR	792.398	218 → 223
						GTEAAGA(OxM)FLEAIP(OxM)SIPPEVK	2290.122	344 → 365
6	sp|P01009|A1AT_HUMAN	46,737	99	23.44	Alpha-1-antitrypsin OS = Homo sapiens	LG(OxM)FNIQ(OxH)CKK	1349.65	224 → 234
						KLSSWVLL(OxM)K	1219.7	234 → 243
						GTEAAGA(OxM)FLEAIP(OxM)SIPPEVK	2290.122	344 → 365

## Data Availability

Not applicable.

## Supplementary Materials

The supporting information can be downloaded at: https://www.mdpi.com/article/10.3390/ijms241713533/s1.

## References

[1] Guéant J.L., Guéant-Rodriguez R.M., Fromonot J., Oussalah A., Louis H., Chery C., Gette M., Gleye S., Callet J., Raso J. (2021). Elastase and exacerbation of neutrophil innate immunity are involved in multi-visceral manifestations of COVID-19. Allergy.

[2] Akgun E., Tuzuner M.B., Sahin B., Kilercik M., Kulah C., Cakiroglu H.N., Serteser M., Unsal I., Baykal A.T. (2020). Proteins associated with neutrophil degranulation are upregulated in nasopharyngeal swabs from SARS-CoV-2 patients. PLoS ONE.

[3] Bhattacharyya C., Das C., Ghosh A., Singh A.K., Mukherjee S., Majumder P.P., Basu A., Biswas N.K. (2021). SARS-CoV-2 mutation 614G creates an elastase cleavage site enhancing its spread in high AAT-deficient regions. Infect. Genet. Evol..

[4] Pandolfi L., Fossali T., Frangipane V., Bozzini S., Morosini M., D’Amato M., Lettieri S., Urtis M., Di Toro A., Saracino L. (2020). Broncho-alveolar inflammation in COVID-19 patients: A correlation with clinical outcome. BMC Pulm. Med..

[5] Cagnone M., Piloni D., Ferrarotti I., Di Venere M., Viglio S., Magni S., Bardoni A., Salvini R., Fumagalli M., Iadarola P. (2019). A Pilot Study to Investigate the Balance between Proteases and α1-Antitrypsin in Bronchoalveolar Lavage Fluid of Lung Transplant Recipients. High Throughput.

[6] Janciauskiene S.M., Bals R., Koczulla R., Vogelmeier C., Köhnlein T., Welte T. (2011). The discovery of α1-antitrypsin and its role in health and disease. Respir. Med..

[7] De Serres F., Blanco I. (2014). Role of alpha-1 antitrypsin in human health and disease. J. Intern. Med..

[8] Brantly M. (2002). Alpha-1 antitrypsin: Not just an antiprotease. Extending the half-life of a natural anti-inflammatory molecule by conjugation with polyethylene glycol. Am. J. Respir. Cell Mol. Biol..

[9] Janciauskiene S., Nita I., Subramaniyam D., Li Q., Lancaster J.R., Matalon S. (2008). Alpha1-antitrypsin inhibits the activity of the matriptase catalytic domain in vitro. Am. J. Respir. Cell Mol. Biol..

[10] Bergin D.A., Reeves E.P., Meleady P., Henry M., McElvaney O.J., Carroll T.P., Condron C., Chotirmall S.H., Clynes M., O’Neill S.J. (2010). α-1 Antitrypsin regulates human neutrophil chemotaxis induced by soluble immune complexes and IL-8. J. Clin. Investig..

[11] Pajdak W., Sieradzak-Fleituch M., Dubin A., Owsiński J. (1991). Alpha-1-antitrypsin and alpha-1-antitrypsin-neutrophil elastase complex in bronchoalveolar lavage fluid of patients with pulmonary diseases (pilot study). Acta Med. Hung..

[12] Fujita J., Bungo M., Hata Y., Nakamura H., Shiotani T., Irino S. (1989). Evaluation of the elastase: Alpha 1-antitrypsin balance in patients with bacterial pneumonia using bronchoalveolar lavage. Nihon Kyobu Shikkan Gakkai Zasshi.

[13] Hirsch J., Elssner A., Mazur G., Maier K.L., Bittmann I., Behr J., Schwaiblmair M., Reichenspurner H., Fürst H., Briegel J. (1999). Bronchiolitis obliterans syndrome after (heart-)lung transplantation. Impaired antiprotease defense and increased oxidant activity. Am. J. Respir. Crit. Care Med..

[14] Yusen R.D., Edwards L.B., Kucheryavaya A.Y., Benden C., Dipchand A.I., Dobbels F. (2014). The registry of the International Society for Heart and Lung Transplantation: Thirty-first adult lung and heart-lung transplant report—2014; focus theme: Retransplantation. J. Heart Lung Transpl..

[15] Oguntuyo K.Y., Stevens C.S., Siddiquey M.N., Schilke R.M., Woolard M.D., Zhang H., Acklin J.A., Ikegame S., Hung C.T., Lim J.K. (2020). In plain sight: The role of alpha-1-antitrypsin in COVID-19 pathogenesis and therapeutics. bioRxiv.

[16] Ritzmann F., Chitirala P., Krüger N., Hoffmann M., Zuo W., Lammert F., Smola S., Tov N., Alagem N., Lepper P.M. (2021). AAT-in-COVID-19 Study Group. Therapeutic Application of Alpha-1 Antitrypsin in COVID-19. Am. J. Respir. Crit. Care Med..

[17] D’Amato M., Vertui V., Pandolfi L., Bozzini S., Fossali T., Colombo R., Aliberti A., Fumagalli M., Iadarola P., Didò C. (2022). Investigating the Link between Alpha-1 Antitrypsin and Human Neutrophil Elastase in Bronchoalveolar Lavage Fluid of COVID-19 Patients. Curr. Issues Mol. Biol..

[18] Li Z., Alam S., Wang J., Sandstrom C.S., Janciauskiene S., Mahadeva R. (2009). Oxidized α1-antitrypsin stimulates the release of monocyte chemotactic protein-1 from lung epithelial cells: Potential role in emphysema. Am. J. Physiol. Lung Cell Mol. Physiol..

[19] Topic A., Milovanovic V., Lazic Z., Ivosevic A., Radojkovic D. (2018). Oxidized Alpha-1-Antitrypsin as a Potential Biomarker Associated with Onset and Severity of Chronic Obstructive Pulmonary Disease in Adult Population. J. Chron. Obstr. Pulm. Dis..

[20] Janciauskiene S. (2020). The Beneficial Effects of Antioxidants in Health and Diseases. Chronic Obstr. Pulm. Dis..

[21] Lechowicz U., Rudzinski S., Jezela-Stanek A., Janciauskiene S., Chorostowska-Wynimko J. (2020). Post-Translational Modifications of Circulating Alpha-1-Antitrypsin Protein. Int. J. Mol. Sci..

[22] Stoller J.K., Aboussouan L.S. (2005). Alpha1-antitrypsin deficiency. Lancet.

[23] Campos M., Lascano J. (2017). Therapeutics: Alpha-1 Antitrypsin Augmentation Therapy. Methods Mol. Biol..

[24] Davies M.J. (2016). Protein oxidation and peroxidation. Biochem. J..

[25] McElvaney O.J., McEvoy N.L., McElvaney O.F., Carroll T.P., Murphy M.P., Dunlea D.M., Ní Choileáin O., Clarke J., O’Connor E., Hogan G. (2020). Characterization of the Inflammatory Response to Severe COVID-19 Illness. Am. J. Respir. Crit. Care Med..

[26] Taggart C., Cervantes-Laurean D., Kim G., McElvaney N.G., Wehr N., Moss J., Levine R.L. (2000). Oxidation of either methionine 351 or methionine 358 in alpha 1-antitrypsin causes loss of anti-neutrophil elastase activity. J. Biol. Chem..

[27] Griffiths S.W., Cooney C.L. (2002). Relationship between protein structure and methionine oxidation in recombinant human alpha 1-antitrypsin. Biochemistry.

[28] Ciaramelli C., Fumagalli M., Viglio S., Bardoni A.M., Piloni D., Meloni F., Iadarola P., Airoldi C. (2017). ^1^H NMR To Evaluate the Metabolome of Bronchoalveolar Lavage Fluid (BALf) in Bronchiolitis Obliterans Syndrome (BOS): Toward the Development of a New Approach for Biomarker Identification. J. Proteome Res..

[29] Smith P.K., Krohn R.I., Hermanson G.T., Mallia A.K., Gartner F.H., Provenzano M.D., Fujimoto E.K., Goeke N.M., Olson B.J., Klenk D.C. (1985). Measurement of protein using bicinchoninic acid. Anal. Biochem..

[30] Yvon M., Chabanet C., Pélissier J.P. (1989). Solubility of peptides in trichloroacetic acid (TCA) solutions. Hypothesis on the precipitation mechanism. Int. J. Pept. Protein Res..

[31] Laemmli U.K. (1970). Cleavage of structural proteins during the assembly of the head of bacteriophage T4. Nature.

[32] Candiano G., Bruschi M., Musante L., Santucci L., Ghiggeri G.M., Carnemolla B., Orecchia P., Zardi L., Righetti P.G. (2004). Blue silver: A very sensitive colloidal Coomassie G-250 staining for proteome analysis. Electrophoresis.

[33] Di Venere M., Fumagalli M., Cafiso A., De Marco L., Epis S., Plantard O., Bardoni A., Salvini R., Viglio S., Bazzocchi C. (2015). Ixodes ricinus and Its Endosymbiont *Midichloria mitochondrii*: A Comparative Proteomic Analysis of Salivary Glands and Ovaries. PLoS ONE.

