# Supplementary material for: Could the Oxidation of α1-Antitrypsin Prevent the Binding of Human Neutrophil Elastase in COVID-19 Patients?
Source: Int J Mol Sci. 2023 Aug 31;24(17):13533. doi: 10.3390/ijms241713533 (PMC10488172; doi:10.3390/ijms241713533)
Supplement: Supplementary file 1 [file ijms-24-13533-s001.zip › ijms-2590175-supplementary.pdf]

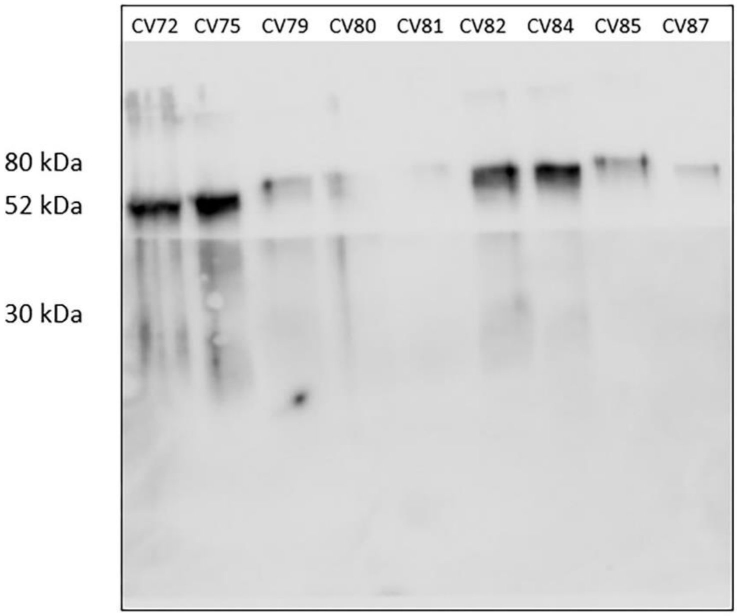

Figure S1. Western Blot of BALf from COVID-19 samples supplemented with exogenous AAT, upon treatment with anti-AAT and anti-HNE antibodies

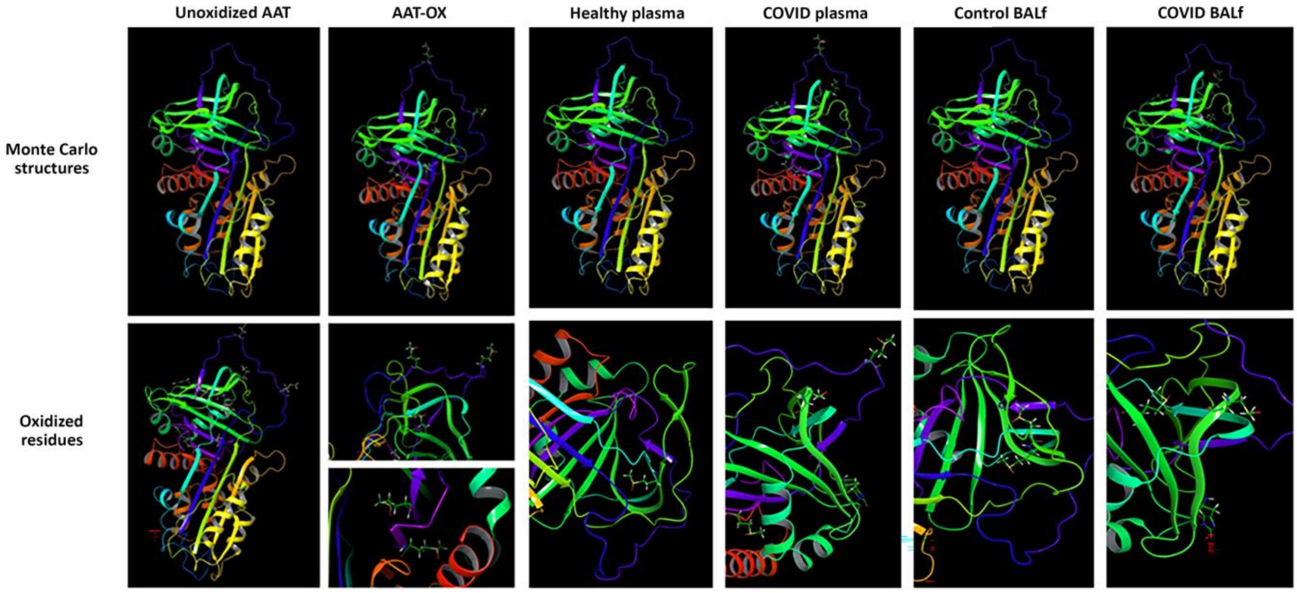

Figure S2. Structures of unoxidized AAT AAT-OX, AAT purified from plasma of healthy individuals, AAT purified from plasma of COVID-19 patients, AAT purified from BALf of controls(left) and aat purified from BALf of COVID-19 patients (from left to right).

Table S1. Energy values determined by Prime for standard unoxidized AAT, AAT-OX and AAT purified from plasma and BALf from controls/COVID 19 patients.

**Structure**	**Prime energy (kcal/mol)**	**Hybrid Monte Carlo (kcal/mol)**
Unoxidized AAT	-13268.5	-15411.4
AAT-OX	-13232.2	-15429.3
COVID plasma	-13370.8	-15511.4
Healthy plasma	-13269.3	-15450.8
COVID BALf	-13372.6	-15532.1
Control BALf	-13282.9	-15485.3
